# The Status of the Antitoxin

**Published:** 1896-01

**Authors:** 


### *EDITORIAL.*

---

The office of the Business Manager of THE JOURNAL is Nos. 311 and 312 Fitten building.
**The editors are not responsible for views expressed by contributors.**

**Articles for publication should reach this office not later than the 15th instant.**

**Address all communications, and make all remittances payable to THE ATLANTA MEDICAL AND SURGICAL JOURNAL, Atlanta, Ga.**

**Reprints of articles will be furnished, when desired, at a nominal cost. Requests for the same should always be made *on the manuscript*.**

---

### THE STATUS OF THE ANTITOXIN.

In connection with the discussion upon diphtheria in the Society Reports of this issue, some remarks upon the present status of the serum treatment will not be out of place.

While it is still too early to foresee with any degree of certainty the position which the antitoxin will ultimately occupy in our therapeutics, enough evidence has accumulated to warrant the assertion that the remedy possesses curative powers for diphtheria far exceeding anything else which has been employed heretofore. Observations and statistics have been so uniformly in favor of this remedy that some have gone so far as to formulate the principle upon which it is based into a general law to the effect: that "whenever an individual, animal or human, has been rendered artificially immune against a certain disease by inoculation with the attenuated toxins elaborated by the bacilli of that disease, the blood serum of this individual may be transferred into another individual, conferring the same immunity upon the latter." Whether Behring's law, as it is called, will be found to conform to the rigid requirements of a law of nature, or prove to be only an expression of a therapeutic principle applicable within certain limits, remains to be seen.

Eulenberg, of Berlin, has reported upon 10,240 cases treated outside of hospitals between October 1, 1894, and March 31, 1895. Five thousand seven hundred and ninety were treated with antitoxin, the remainder by other methods. The death rate among those who received the antitoxin was 9.5 per cent.; among those who did not it was 14.6 per cent. Of those under two years who received the serum treatment 21.7 per cent. died; for the same age those who did not receive this treatment 39.5 per cent. died. Eulenberg concludes from his observations that the "serum treatment of diphtheria appears the most efficacious yet known, and physicians will find it their most reliable weapon against the disease." Zabolotnui reports 109 cases treated in the district of Podolia, Russia, with a mortality of 12.8 per cent.; in former epidemics the mortality had averaged 48 per cent. One hundred cases lately treated in the Children's Hospital in Moscow showed a death rate of 19. For the four years previous the mortality had averaged 47 per cent. per year.

Von Ranke, of Munich, says that up to September, 1894, the yearly mortality of cases which he had treated had averaged 52 per cent. Since this date, when the serum method was begun, the mortality has been 17.7 per cent. Baginsky, of Berlin, reports a similar diminution in his mortality from 41 to 15.6 per cent. Kurth, of Bremen, has had a death rate of 6.8 per cent. among patients treated with the serum, and 24 per cent. among those treated without it. In one of the Berlin hospitals last year the mortality under the serum treatment for four or five months had been reduced to 15.6 per cent. For two months (August and September, 1894,) the supply of serum was exhausted and the mortality under other methods rose to 48.4 per cent. When the serum was resumed, the former death rate of about 15 or 16 per cent. was again observed for the next six months.

It would be easy to multiply reports such as the above from some of the largest clinics of Europe. These reports are almost all of one kind, and agree that the serum treatment has reduced the death rate from 40 or 50 per cent. to about 15 per cent., that fewer cases develop laryngeal diphtheria, and fewer require intubation or tracheotomy.

In our own country valuable contributions to the literature and knowledge of this subject have lately been made by Dr. W. H. Welch, of Baltimore, and Dr. Edwin Rosenthal, of Philadelphia. Dr. Welch's paper appeared in the *Bulletin* of the Johns Hopkins Hospital for July and August, and was based upon the author's remarks upon this subject before the Association of American Physicians, in Washington, in May. This paper is the result of thoughtful and unprejudiced study, and is worthy of a careful reading by every one. Dr. Welch presents a synopsis of eighty-two reports, embracing 7,166 cases of diphtheria treated with anti-toxin. These cases include none of the 10,000 of Eulenberg's report, referred to above. Of these 7,166 cases, 17.3 per cent. died. Five thousand four hundred and six of these cases were gathered from certain hospitals and localities where the mortality, under former methods of treatment, had been 42 per cent.; under the serum treatment for these 5,406 cases, the number of deaths was 1,008, or 18.6 per cent. Dr. Welch says that this striking difference cannot be accounted for by assuming that these cases all represented a mild type of the disease.

From a study of these 7,000 cases, Dr. Welch is of the opinion that the anti-diphtheritic serum is without doubt a specific curative agent, and that it is the duty of every physician to use it. He thinks that these studies in immunization and the resulting discoveries mark an epoch in the history of medicine.

Dr. Rosenthal, of Philadelphia, after a large experience, says that he regards the antitoxic serum as a cure for pure and simple diphtheria. "When used before the diphtheria toxins have so invaded the tissues as to destroy them, antitoxin is a specific." This gentleman has published in the *Medical and Surgical Reporter* (November 2 and November 9) a collective investigation of 222 cases treated in hospitals and private practice by twenty-one physicians. Of this number, thirteen died—a mortality of not quite 6 per cent. In this author's own experience, he had had eighty-four cases, with twelve deaths—a mortality of 14.3 per cent. Dr. Rosenthal's conclusions are strongly favorable to the use of antitoxin. In the Boston City Hospital there have been 113 cases treated between July 1 and November 1. "There were ninety recoveries and twenty-three deaths. Of the twenty-three deaths, fifteen were hopeless cases when admitted. Of the ninety-eight cases that received seasonable treatment, only eight died—a mortality of about 8 per cent. Formerly the mortality had been 50 per cent." For four years prior to 1895 the average yearly mortality from diphtheria in New York City had been about 34 per cent.; for this year it has been 19 per cent. Commenting upon these figures, the President of the New York Board of Health, Dr. Chas. G. Wilson, says:

"This large reduction in the mortality rate from diphtheria and croup, for the first three quarters of 1895, is attributed mainly by the medical officers of this department to the introduction and use of diphtheria antitoxin, and if the remedy had been generally or universally employed the reduction in the mortality rate would doubtless have been larger."

"All theoretical considerations," Virchow says, "must give way to the brute force of these figures."

In this country, perhaps, the most vigorous opponent of the serum treatment has been Dr. J. E. Winters, of New York, a gentleman whose position and prominence certainly entitle his opinions to just consideration. In his remarks before the New York Academy of Medicine, last April, he stated that in his service in the Willard Parker Hospital he had seen no good results from the treatment; that the mortality had not been reduced, and that the complications and sequelæ of the disease had not been prevented or mitigated. These remarks have lately been refuted by Dr. W. H. Park, also physician to the same hospital, and his paper (*Boston Medical and Surgical Journal,* September 12,) agrees, in the main, with the usual reports made by others.

Now, if such figures as I have quoted above represented only the observations of a few men and a few cases, their value for purposes of scientific deduction would be only trifling; but when it is remembered that we now have the reports of many hundreds of cautious and truth-seeking observers upon many thousands of patients, and that these reports show a reduction in the death rate from 40 or 50 per cent. to about 18 or 20 per cent., we think it is impossible for an impartial mind to resist the evidence. The *University Medical Magazine* (November) says editorially:

"Nearly a year has elapsed since the antitoxin treatment of diphtheria has been at the service of more than a few favored investigators, and by this time a sufficient number of cases has accumulated upon which a fair estimate of the value of the healing serum can be based. During the last year there have appeared in the various medical journals reports of the results obtained with the serum treatment in more than 15,000 cases, representing all ages and all degrees of severity, and after a careful consideration of this great mass of testimony, it must be admitted that we have in antitoxin a remedy for diphtheria far superior to any known heretofore, and one which should be employed at the earliest possible moment after the recognition of the disease."

In the last edition of Prof. Osler's work on "Practice," this distinguished author and clinician says that "in favorable cases the effects of the serum are seen in a marked amelioration of both local and general symptoms. The swelling in the fauces subsides, the membrane disappears, the temperature falls, the pulse becomes slower, and the general condition of the patient is improved in every way. While a much larger number of cases must be treated before a final judgment can be reached, meanwhile the antitoxin treatment should be adopted in cases of true diphtheria."

The *Therapeutic Gazette* says editorially (November 15):

"As the months go by and the antitoxin treatment of diphtheria is more and more fully tested, it becomes evident that the early reports of an encouraging nature were well founded, and although one fallacious argument after another has been discovered, the radical principle underlying this method of treatment still remains not only intact, but also stronger in theory and practice than ever before."

